# Involvement of the acromion in cases of distal clavicular osteolysis

**DOI:** 10.1007/s00256-025-05014-0

**Published:** 2025-08-18

**Authors:** Aishwarya Gulati, Blaire Adler, Jeffrey A. Belair

**Affiliations:** 1 Department of Radiology, Thomas Jefferson University Hospital, 132 S. 10th Street, 10th Floor, Philadelphia, PA 19107 USA; 2 Sidney Kimmel Medical College, Thomas Jefferson University, 1025 Walnut Street, Suite 100, Philadelphia, PA 19107 USA

**Keywords:** Clavicle, Acromion, Distal clavicular osteolysis (DCO), Acromioclavicular (AC) joint, MRI

## Abstract

**Objective:**

Distal clavicular osteolysis (DCO) is a commonly encountered cause of shoulder pain resulting from repetitive overuse or antecedent trauma, classically described in young male weightlifters. We propose a variant of DCO in which osteolysis spans the acromioclavicular joint, involving both the anterior acromion and the distal clavicle.

**Materials and methods:**

A retrospective PACS query identified patients with DCO on shoulder MRIs performed at ≥ 1.5 T over a 1-year period. After inclusion/exclusion criteria were applied, each case was reviewed in a blinded fashion to assess for additional findings of osteolysis involving the acromion. Demographics and patient questionnaire data were recorded and analyzed for statistical significance between groups.

**Results:**

A total of 128 cases of DCO were identified in 127 patients (93 males). Mean age was 39.5 years (SD 11.3 years). Average symptom duration was 409 days (13.4 months). Per questionnaires, 45.3% had a history of antecedent trauma, 62.5% reported lifting weights, 38.3% reported overhead sports, and 32.0% reported repetitive activities. Of the 128 cases, 42 (32.8%) had additional findings of osteolysis involving the acromion. Acromial involvement was seen more commonly in males (*p* = 0.049). Other than sex, maximum bench press weight was the only statistically significant factor associated with acromial involvement (*p* = 0.027).

**Conclusion:**

We identified a variant of DCO with osteolysis involving the acromion in addition to the distal clavicle. Other than male sex, maximum bench press weight was the only significant factor associated with acromial involvement, suggesting that increased load bearing may contribute to more extensive osteolysis.

## Introduction

The acromioclavicular joint (ACJ) is a small planar (gliding) diarthrodial synovial joint between the distal clavicle and anterior acromion. It is one of three articulations that, along with the sternoclavicular and glenohumeral joints, connects the upper extremity to the axial skeleton and allows for movement of the upper limb [1].

DCO is manifest by painful bone resorption with intense, confluent bone marrow edema, loss of cortical margins, and osseous erosion of the distal end of the clavicle. DCO may be secondary to repetitive overuse and stress at the joint, classically described in weightlifters and young athletic males. It may also be seen in the posttraumatic setting, with symptoms reported anywhere from weeks to months after the initial insult [1]. The overall incidence of DCO and the proportion of repetitive overuse versus posttraumatic etiologies remains unclear, but DCO has been reported in about 6–10% of patients after ACJ injury [1, 2]. A study of over 7000 patients reported a prevalence of atraumatic DCO as 5%, of which only 9% were female [3]. The incidence has been reported as high as 28% in weightlifters [4].

The pathophysiology of DCO is thought to be similar following both acute trauma and repetitive overuse, whereby the insult leads to subchondral microfracture, increased osteoclastic activity, and bone resorption. With continued biomechanical stress across the joint, repeated attempts at healing subsequently result in increased osteoblastic activity, capsular and synovial inflammation, cartilage degeneration, and eventual fibrosis [5, 6].

While post-traumatic DCO does not have an age predilection, atraumatic DCO is classically seen in a younger, typically male population. Overhead athletes and weightlifters are classically affected [3, 6]. The initial complaint is often an insidious dull aching pain at the joint, exacerbated by movement, and may be bilateral in up to 20% of cases [1, 6]. Pain may also radiate up the neck or down the arm, which can occasionally cause misattribution of symptoms to the cervical spine or glenohumeral joint, resulting in delayed diagnosis. DCO may also be misdiagnosed as ACJ osteoarthritis (and occasionally, vice versa). Misdiagnosis can lead to delay in appropriate treatment—specifically, immobilization and/or removal of any inciting repetitive overuse activities [6].

**Fig. 1:**
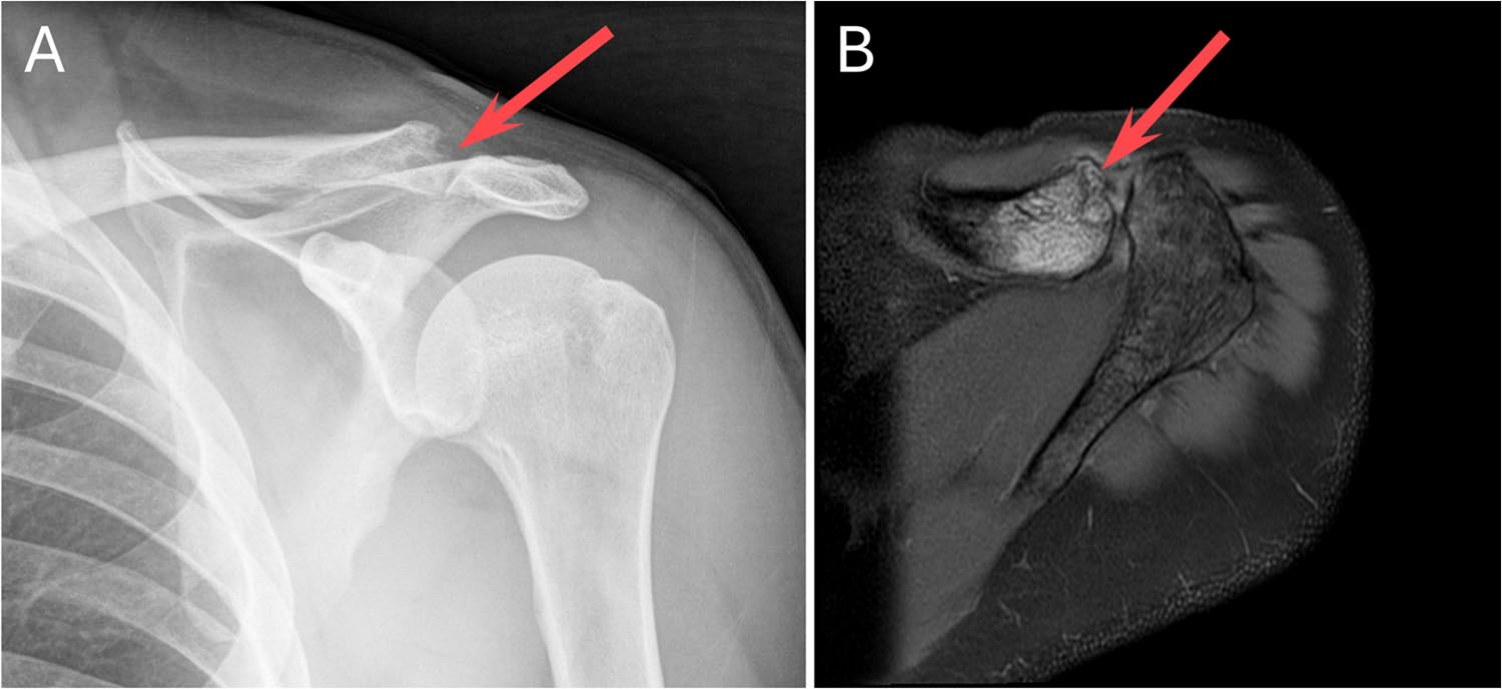
**A** Externally rotated AP shoulder radiograph demonstrates classic X-ray findings of DCO, with osseous resorption of the distal clavicle (arrow). **B** Axial PD fat-suppressed MR image of the shoulder demonstrates classic MRI findings of DCO, including intense bone marrow edema and osseous resorption of the distal clavicle (arrow)

Radiographs have poor sensitivity in the initial stages of disease. In later stages, loss of cortical margins and subchondral bone resorption/erosion of the distal clavicle can be seen (Fig. 1) [1, 2]. The Zanca view, an anteroposterior projection with the X-ray beam tilted 15–30° cephalad, can optimize visualization of the ACJ [1]. While not commonly used, ultrasound can demonstrate distal clavicular irregularity and hyperemia at the ACJ [7]. MRI is more sensitive and of particular use in the early stages of disease. Initially, intense bone marrow edema in the distal clavicle on fluid-sensitive sequences indicates osseous stress reaction, with additional findings including capsular hypertrophy and edema, joint effusion, and periarticular soft tissue edema. Subsequently, subchondral bone resorption/erosion with loss of cortical margins and hypointense marrow signal on T1-weighted imaging is seen [1]. Discrete subchondral fracture lines in the distal clavicle have also been reported [3, 8].

DCO is usually treated conservatively with non-steroidal anti-inflammatory drugs, ice, rest from causative activities, and physical therapy [9]. Intra-articular corticosteroid injections can be considered to help manage acute symptoms but could result in delayed healing and should be used with caution. Recently, the use of platelet-rich plasma has been reported with some success in patients who fail to respond to conservative therapy [9, 10]. As a last resort in refractory cases, resection of the distal clavicle can be performed [9].

While DCO has classically been described as isolated to the distal clavicle, we hypothesize that a variant may be seen affecting both the distal clavicle and the anterior acromion, with bone marrow edema and osteolysis spanning the ACJ. This is analogous to the resorptive changes that may be seen at the pubic symphysis in severe osteitis pubis. Primary differential considerations for this appearance would include various inflammatory arthropathies, septic arthritis, and hyperparathyroidism. Thus, recognizing this variant of DCO can have important clinical implications, as this condition requires no further workup and is potentially amenable to conservative treatment with activity modification.

## Materials and methods

### Patient selection

This study was performed in accordance with ethical principles outlined in the Declaration of Helsinki and carried out in compliance with Health Insurance Portability and Accountability Act (HIPAA) regulations. The study was approved by our Institutional Review Board (IRB); a waiver of informed consent was granted by the IRB. A retrospective study was conducted to collect a DICOM data set of shoulder MRIs performed at our institution, which included imaging findings of DCO in the report impression. Our PACS database was queried to identify patients imaged over a single year (2023) with a diagnosis of DCO on shoulder MRI reports using the search strings “distal clavicular osteolysis,” “osteolysis,” and “DCO.”

Inclusion criteria were studies with a diagnosis of DCO performed using a standardized shoulder MRI protocol on a ≥ 1.5 Tesla scanner, including the following sequences: axial PD fat-suppressed, coronal STIR, coronal T1, sagittal T2, and sagittal T2 fat-suppressed. Exclusion criteria were the presence of significant background ACJ osteoarthritis, poor image quality, and studies that did not adhere to our standardized imaging protocol. Significant ACJ osteoarthritis was defined as any degree of osteoarthritis graded greater than “mild” on the initially issued standardized report (i.e., “moderate” or “severe”) or upon further review by the blinded musculoskeletal radiology fellow and attending. Patients who did not complete a standard pre-imaging shoulder questionnaire were also excluded. Patients with other shoulder soft tissue pathology, such as rotator cuff tendinopathy or labral tears, were not excluded.

### Data set collection

**Fig. 2:**
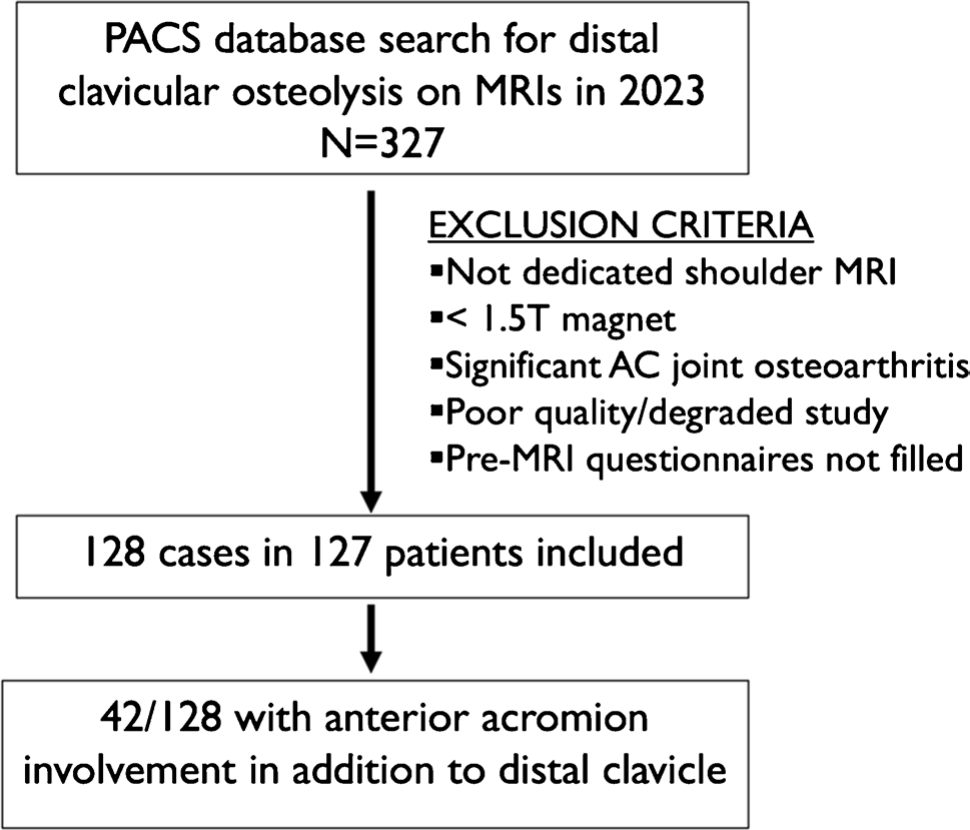
Patient selection criteria

A fourth-year medical student and a musculoskeletal radiology fellow identified 327 shoulder MRIs that were performed in 2023 with a diagnosis of DCO in the impression of the report. After applying all inclusion and exclusion criteria, 128 MRIs of 127 patients were included (Fig. 2). Demographic data, including patient age and sex, were collected. Clinical information, including duration of symptoms, hand dominance, side of involvement, history of trauma, repetitive activity, overhead sports, weightlifting, and maximum bench press weight, were collected via pre-imaging questionnaires completed by all patients.

### Retrospective study

**Fig. 3:**
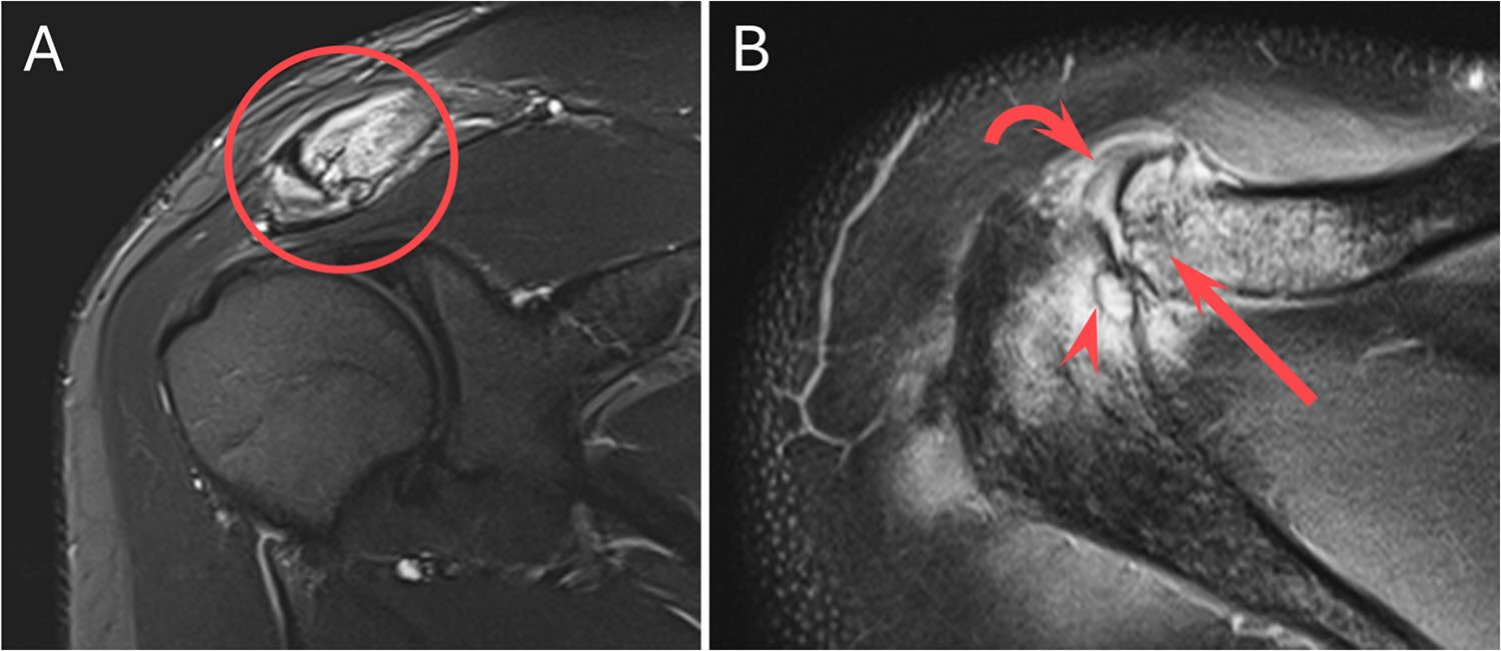
A 28-year-old male presenting a few months after a fall at work. Coronal STIR (**A**) MR image of the shoulder demonstrates intense bone marrow edema and resorption of the distal clavicle, consistent with DCO (circled). Axial PD fat-suppressed (**B**) MR image demonstrates similar findings of DCO, including intense bone marrow edema, resorption, and cystic change of the distal clavicle (arrow), capsular thickening and edema (curved arrow), and additional findings of osteolysis involving the anterior acromion (arrowhead). Note the lack of significant bony productive change, which would typically be seen with osteoarthritis

**Fig. 4:**
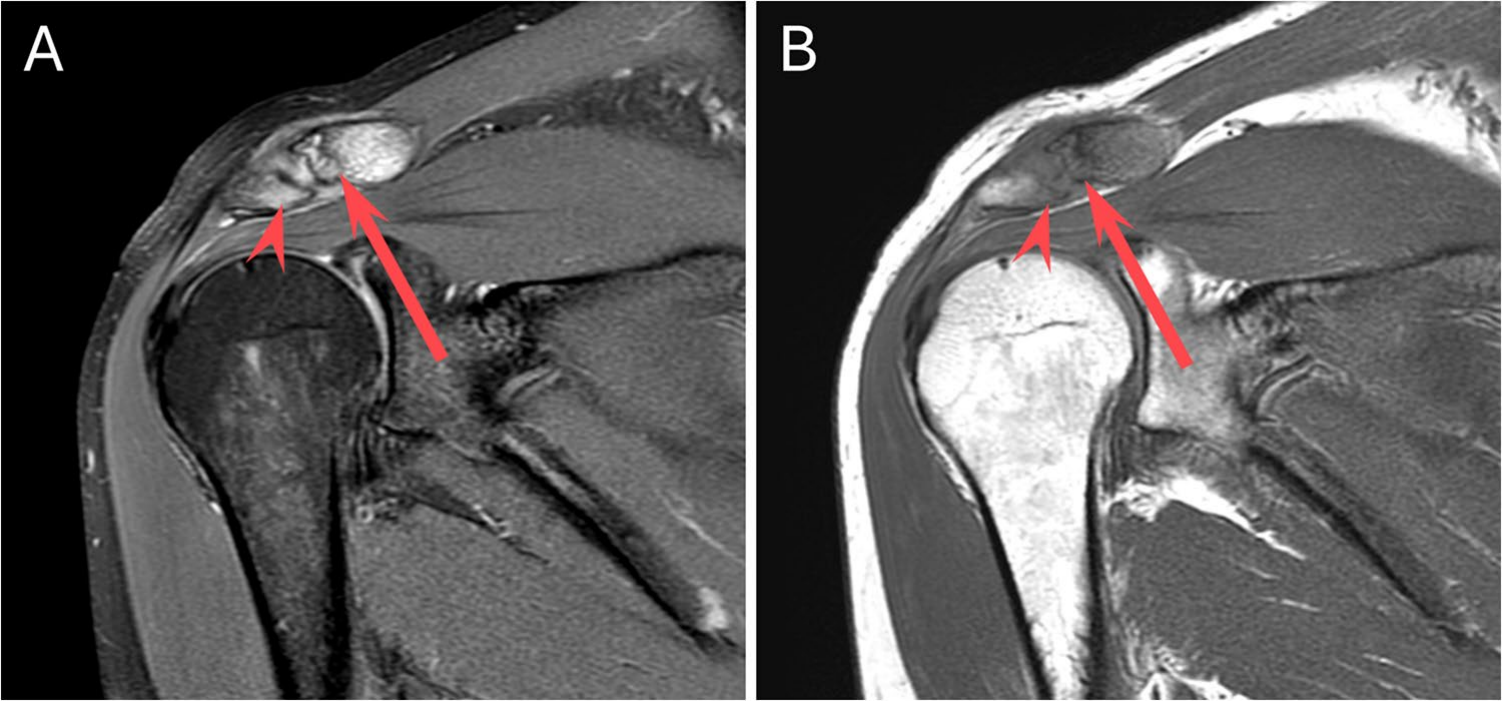
A 46-year-old delivery truck driver with acute-on-chronic right shoulder pain related to overuse. Coronal STIR (**A**) and coronal T1 (**B**) MR images demonstrate findings of osteolysis both in the distal clavicle (arrow, **A** and **B**) and in the anterior acromion (arrowhead, **A** and **B**)

**Fig. 5:**
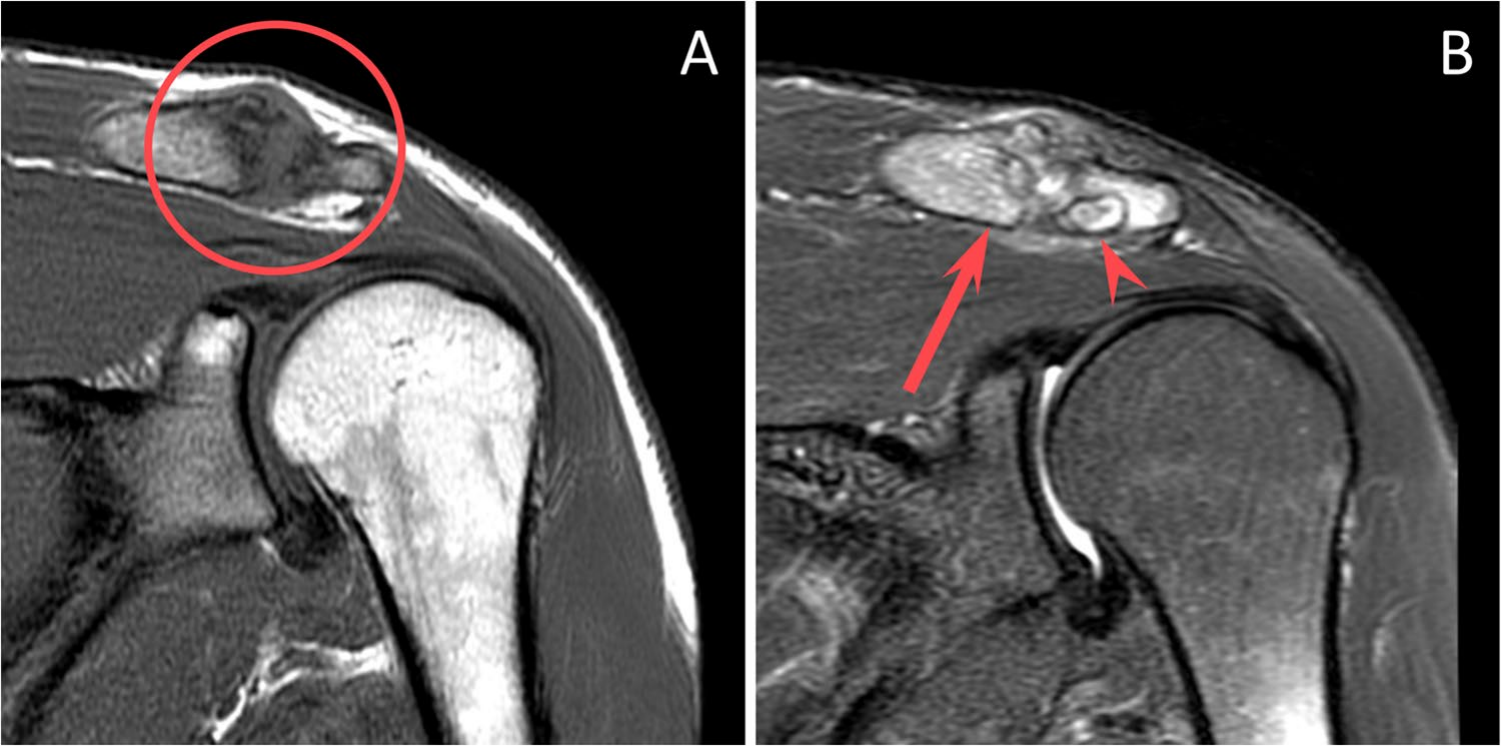
A 32-year-old weightlifter with ongoing shoulder pain for 8–9 months. Coronal T1 (**A**) MR image demonstrates osteolysis spanning the acromioclavicular joint, with bone resorption, loss of cortical margins, and T1-hypointensity (circled). Coronal STIR (**B**) MR image demonstrates intense bone marrow edema with osseous resorption and cystic change in the distal clavicle (arrow) and anterior acromion (arrowhead). Note that there is a suggestion of mild underlying acromioclavicular joint osteoarthritis, with small marginal osteophyte formation at the superior margin of the joint

Each MRI was re-reviewed by a musculoskeletal fellow and a musculoskeletal radiology attending (8 years experience) in consensus, blinded to clinical history and the originally issued report. Findings typical for DCO present on each MRI included bone marrow edema and osseous resorption/erosion involving the distal clavicle (see Fig. 1). Any additional, similar findings involving the anterior acromion were recorded (Figs. 3, 4, and 5).

**Fig. 6:**
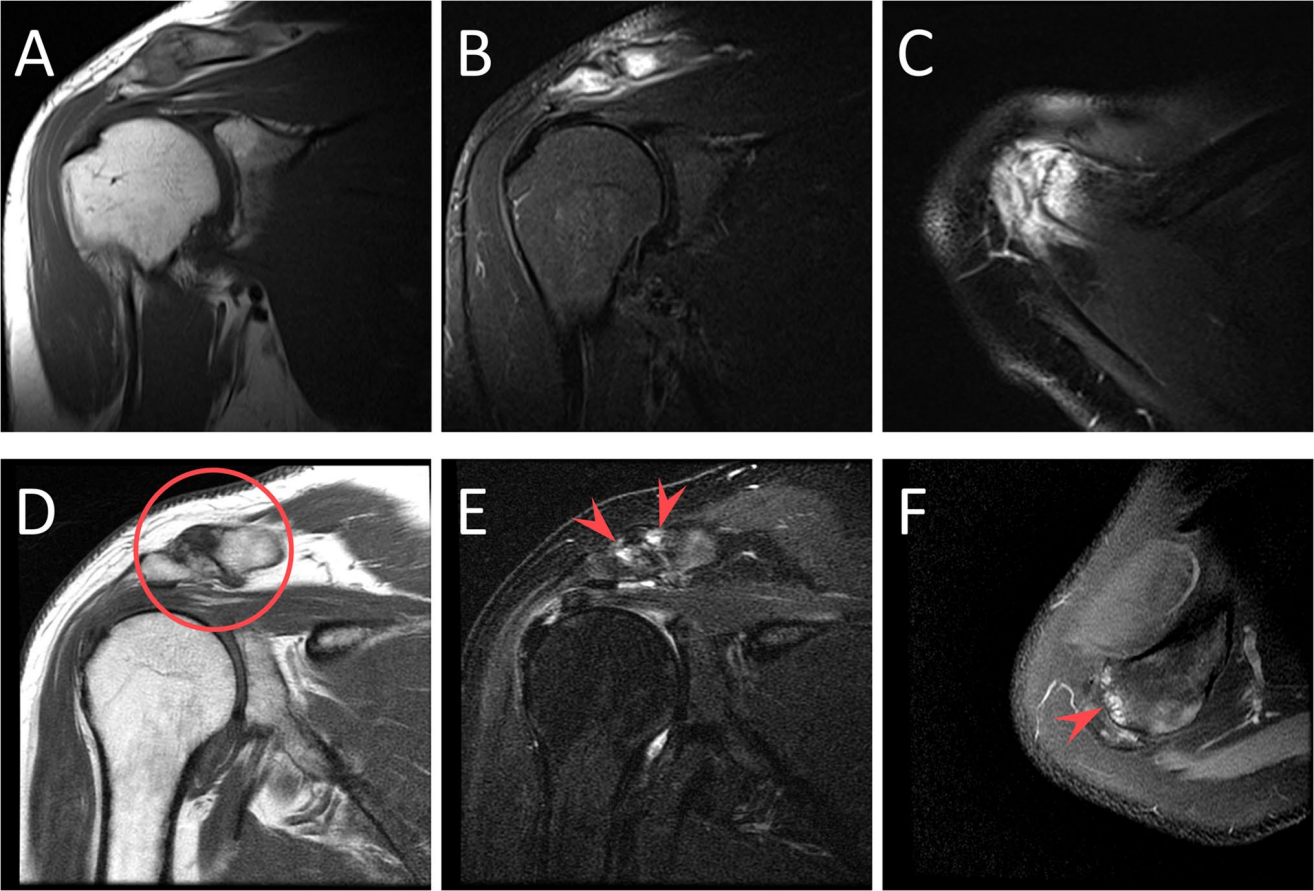
Coronal T1 (**A**), coronal STIR (**B**), and axial PD fat-suppressed (**C**) MR images demonstrating findings of post-traumatic distal clavicular osteolysis with involvement of the anterior acromion in a 46-year-old with shoulder pain for 2 months after a fall with hyperextension of the arm (**A**–**C**). Contrast this case with findings of severe osteoarthritis in a 65-year-old with chronic shoulder pain (**D**–**F**), in which coronal T1 (**D**), coronal STIR (**E**), and axial PD fat-suppressed (**F**) MR images demonstrate joint space narrowing with marginal osteophytes (circled, **D**) and subchondral cystic change without significant associated bone marrow edema (arrowheads, **E** and **F**)

Osteolysis was differentiated from ACJ osteoarthritis by the following criteria: subchondral bone marrow edema disproportionate to the degree of underlying osteoarthritis (only cases of mild or no ACJ osteoarthritis were included in the study); small or no marginal osteophytes; osseous resorption/erosion with poorly defined cortical margins; replacement of normal marrow signal (i.e., hypointensity) on T1-weighted imaging. While osseous erosions and subchondral cysts may be indistinguishable on MRI, subchondral cystic change in the context of osteoarthritis typically lacks the additional findings of intense confluent bone marrow edema and loss of cortical margins and often demonstrates coexistent marginal osteophytes. Figure 6 depicts an example of DCO with anterior acromial involvement compared to a case of severe ACJ osteoarthritis.

### Statistical analysis

The total incidence of anterior acromial involvement in cases of DCO was calculated. The characteristics of the group with anterior acromial involvement were compared with those of the group with classic DCO in which only the distal clavicle was affected. Statistical analysis was performed using IBM® SPSS® Statistics. Binomial logistic regression was performed comparing the age, sex, duration of symptoms, hand dominance, side of involvement, history of trauma, repetitive activity, overhead sports, weightlifting, and maximum bench press weight in both groups. Cases in which data was partially unavailable were excluded from relevant calculations.

## Results

A total of 327 radiology reports mentioned DCO in the impression of MRIs reported in 2023. After exclusion of 199 cases, 128 cases met inclusion criteria in 127 patients (one male patient had imaging of both shoulders). Of the included patients, 93 were male and 34 were female. The mean patient age at time of imaging was 39.5 years (SD 11.3 years, range 16–70 years). Average symptom duration before MRI was 409 days (13.4 months) (*n* = 110; duration not reported in 18 cases). On pre-imaging shoulder questionnaires, 45.3% had a history of antecedent trauma, 62.5% reported lifting weights, 38.3% reported overhead activities or sports, and 32.0% reported other repetitive movements.

Of the 128 cases included, 42 (32.8%) had findings of osteolysis involving the anterior acromion in addition to the distal clavicle, with one male having this finding in both shoulders. Of the patients with acromial involvement, 81% were male, compared to 68% in cases with clavicular involvement only (*p* = 0.049). The mean reported maximum bench press weight in patients with acromial involvement was 267.6 pounds (*n* = 21), compared to 206.25 pounds in the group without anterior acromial involvement (*n* = 47). Other than sex, this was the only statistically significant factor associated with acromial involvement (*p* = 0.027). There was no statistically significant difference (*p* < 0.05) between the patients of each group with respect to history of trauma, weightlifting, playing overhead sports, repetitive motion, or duration of symptoms. There was a longer average duration of symptoms in patients with acromial involvement, which was not statistically significant. Two patients had an os acromiale detected on MRI, both in the group with anterior acromial involvement. Two patients had a history of prior rotator cuff repair, and one patient had a history of prior labral repair, all in the group with anterior acromial involvement. None of the patients with osteolysis isolated to the distal clavicle had a history of prior shoulder surgery. Results are displayed in Tables 1 and 2.

**Table 1:** Overall summary of patient characteristics

Age	39.5 years (SD 11.3)
M:F	93:34
Symptom duration (days)	409 (13.4 months, *n* = 110)
Antecedent trauma	45.3%
Lifting weights	62.5%
Overhead activity/overhead sports	38.3%
Other repetitive movements	32.0%

**Table 2:** Comparison of population characteristics between groups

	Overall	Clavicle only	Acromion + clavicle	Sig
Number of cases	128	86	42	-
Male to female ratio	93:34 (73% male)	59:27 (68% male)	34:7 (81% male)	0.049
Age (years)	39.5 (SD 11.3)	39.6 (SD 10.1)	39.5 (SD 13.3)	0.678
Duration of symptoms (days)	409 (13.4 mos, *n* = 110)	371 (12.2 mos, *n* = 76)	493 (16.2 mos, *n* = 34)	0.905
Right:left side	75:53	51:35	24:18	0.786
Dominant side	55 (58.5%, *n* = 94)	33 (55.0%, *n* = 60)	22 (64.7%, *n* = 34)	0.976
History of trauma	58 (45.3%)	38 (44.2%)	20 (47.6%)	0.533
History of weightlifting	80 (62.5%)	53 (61.6%)	27 (64.3%)	0.490
Maximum bench press weight (lbs)	225 (*n* = 68)	206.3 (*n* = 47)	267.6 (*n* = 21)	0.027
History of overhead sports	49 (38.3%)	30 (34.9%)	19 (45.2%)	0.659
Other repetitive motion	41 (32.0%)	28 (32.6%)	13 (31.0%)	0.895
Os acromiale	2 (1.6%)	0 (0.0%)	2 (4.8%)	-
Prior shoulder surgery	3 (2.3%)	0 (0.0%)	3 (7.1%)	-

## Discussion

DCO, often referred to as “weightlifter’s shoulder,” predominantly affects individuals engaged in repetitive upper extremity activities involving heavy loading or stress across the ACJ, particularly athletes and manual laborers [11]. It is most commonly reported in weightlifters, with a higher prevalence among those who regularly perform bench press exercises, overhead presses, or similar exercises that stress the ACJ [3, 12]. Higher frequency and intensity of bench pressing, as well as longer duration of bench pressing, are known risk factors for developing DCO [12].

DCO has been classically reported in young- to middle-aged males due to the demographic distribution of weight training and physically demanding occupations, and in other populations subjected to chronic stress or trauma to the ACJ [3, 9]. With increasing recognition of the importance of muscle mass on overall health and emphasis on strength training, there has been a gradual rise in the popularity of weightlifting across all ages and in women [13]. There has been a concomitant rise in weightlifting-associated injuries, which is likely to continue as individuals become increasingly more health-conscious [12, 13].

MRI is the study of choice for demonstrating findings of DCO, including bone marrow edema, osseous resorption/erosion, and pericapsular edema. While reciprocal edema in the anterior acromion has been documented in patients with DCO [8], our study demonstrates a distinct variant in which resorptive changes are seen at the anterior acromion in addition to the distal clavicle. On analysis of demographic data and activity patterns, this was more commonly seen in males and in patients with greater bench press loads. We suspect this represents a more advanced stage in the natural history of the disease, associated with a higher intensity of weightlifting causing increased stress upon the joint. This hypothesis aligns with the widely recognized association between weightlifting and the development of DCO and helps explain the trend of longer symptom duration in patients with anterior acromial involvement. Previous research studies have identified weightlifting as a significant risk factor for DCO, particularly when the activity involves greater intensity, higher frequency, or longer durations of bench pressing [12, 14]. These observations underscore the mechanical burden that such exercises impose upon the ACJ, potentially accelerating the onset or progression of DCO in susceptible individuals. It is interesting that the two cases in which there was an os acromiale and three cases in which there was a history of prior shoulder surgery were all in the group with anterior acromial involvement. It is possible that destabilization or alteration of the normal biomechanics at the ACJ may predispose to more extensive osteolysis.

Our study, although small in size, has implications for diagnosis, treatment, and management of these patients. Bone marrow and resorptive changes spanning the ACJ in an otherwise healthy, active individual may be mistaken for septic arthritis or an inflammatory arthropathy, leading to unnecessary medical workup and inappropriate treatment. Inflammatory and crystalline arthropathies such as rheumatoid arthritis and gout, septic arthritis, or hyperparathyroidism can cause erosion, bone marrow edema, and periarticular inflammation about the ACJ with a similar imaging appearance [15]. The treatment for these pathologies is varied, making accurate diagnosis critical. The diagnosis of DCO, and our proposed variant, can be confidently made with the appropriate clinical history and characteristic imaging findings on MRI. DCO is usually a self-limited disease when identified early and treated with conservative measures. Activity modification plays a pivotal role in alleviating symptoms and preventing disease progression. During its early stages, DCO does not contribute to joint instability, making timely intervention particularly beneficial. However, if diagnosis and treatment are delayed or the provoking insult is not removed, this may result in foreshortening of the distal clavicle with ACJ widening, instability, and eventual osteoarthritis [3, 6].

Our study has several limitations, the most significant being that it was performed as a retrospective analysis at a single institution, lacking a normal control group and reference standard. Follow-up imaging and clinical notes were not reliably available in our study population, and therefore, we could not assess if patients responded appropriately to conservative treatment. However, none of the included patients returned to our institution for image-guided ACJ aspiration or biopsy. Our study is also dependent upon patient-reported data and thus limited by missing data points. Although our findings suggest that maximum bench press weight may influence involvement of the anterior acromion, other associations could be further elucidated in a larger, more diverse patient population data set. This could help confirm if anterior acromial involvement represents a more severe or advanced form of DCO. Likewise, imaging and clinical follow-up after appropriate treatment would help confirm a biomechanical etiology and determine if this variant has a higher predilection for development of ACJ instability and/or osteoarthritis.

While we did exclude cases in which there was significant (i.e., more than mild) pre-existing ACJ osteoarthritis, cases in which there were other concomitant shoulder findings, such as rotator cuff tendinopathy or labral tears, may have confounded our results. However, in routine clinical practice, DCO often coexists with other shoulder pathology, particularly in older individuals. Although images were reviewed by consensus of a musculoskeletal radiology fellow and attending, which may introduce consensus bias, it is important to note that all included cases had already been initially reported by a radiologist within our musculoskeletal division. In other words, patients had already been independently diagnosed with DCO on MRI by a subspecialty-trained musculoskeletal radiologist (nine interpreting radiologists with 5–30 years experience). Many of the issued reports acknowledged involvement of the acromion, though the verbiage and descriptions were variable. We hope that introducing this variant into the literature will help improve reporting in such cases.

In cases of DCO caused by mechanical overuse or repetitive microtrauma, bone marrow edema in the distal clavicle may precede findings of bone resorption and cystic change. In such cases, we advise reporting this as osseous stress reaction, which may be a precursor lesion to true osteolysis. Thus, when looking at this specific variant of DCO with involvement of the acromion, we chose to focus on cases in which there was true osteolysis of the anterior acromion, not simply reciprocal bone marrow edema. In a previously published study examining shoulder MRIs with DCO, 22/36 patients (61%) had additional mild bone marrow edema in the acromion, which was subjectively less intense than the distal clavicle [8]. Another study examining DCO excluded cases in which there was any edema in the acromion entirely [3]. Thus, while it has been reported that disproportionate acromial bone marrow edema may co-occur with DCO, the finding of acromial osteolysis has not yet been presented. Comparison can be drawn to osteitis pubis in the pelvis, in which excessive mechanical load at the pubic symphysis results in osseous stress reaction and clinical symptoms of athletic pubalgia. In more advanced cases, osteitis pubis may also show findings of bone resorption/erosion with osteolysis and cyst-like changes spanning the symphysis, which may be symmetric or asymmetric [16, 17]. Certainly, intrinsic biomechanical differences between the ACJ and pubic symphysis exist, which likely helps explain the variabilities in imaging appearance and patterns of involvement.

Despite these limitations, our data strongly suggest that DCO can have a varied imaging appearance with involvement of the anterior acromion. This variant of DCO should be suggested when characteristic imaging findings are seen in the appropriate clinical context. Patients with DCO typically respond well to conservative treatment, and delayed clinical diagnosis can lead to potential complications such as ACJ osteoarthritis or instability. If clinical history is insufficient or unavailable, or if there is significant underlying osteoarthritis, one may consider other differential diagnoses such as degenerative, inflammatory, and crystalline arthropathies, septic arthritis, or hyperparathyroidism. Further studies may benefit from a more stringent clinical arm and quantifying involvement of the acromion.

## Conclusion

We identified a variant of DCO with resorptive changes involving the anterior acromion in addition to the distal clavicle, which more commonly affected males. Other than sex, maximum bench press weight was the only statistically significant factor associated with acromial involvement, suggesting that increased load bearing may contribute to more extensive osteolysis. Patients with acromial involvement also showed a trend toward longer duration of symptoms, though a larger patient population is likely needed to further elucidate other associations. With an increasing health-conscious population engaging in various physical training activities, including weightlifting, it is likely that this finding will be seen more commonly. It is also likely that with increased awareness of this variant, it will be recognized and reported with more frequency.

## Data Availability

De-identified data supporting this study are available from the corresponding author upon reasonable request.
